# Comorbidity severity adjusted model for predicting mortality in hospitalized patients with COPD

**DOI:** 10.1371/journal.pone.0348191

**Published:** 2026-05-26

**Authors:** Ji Hyeon Seo, Ji Hye Lim

**Affiliations:** 1 Department of Health Science, Dong-A University, Busan, Korea; 2 Department of Health Care Science, Dong-A University, Busan, Korea; Niigata University of Health and Welfare: Niigata Iryo Fukushi Daigaku, JAPAN

## Abstract

**Background:**

Chronic obstructive pulmonary disease (COPD) is a leading cause of morbidity and mortality worldwide, ranking eighth in disability-adjusted life years (DALYs). Patients with COPD frequently exhibit multimorbidity—including cardiovascular disease, cerebrovascular disease, diabetes, and lung cancer. Accordingly, the Charlson Comorbidity Index (CCI) is widely used as a standard tool for comorbidity adjustment; however, as a general-purpose index, it may not fully capture disease-specific patterns of multimorbidity, underscoring the urgency for tailored approaches. Therefore, we aimed to construct a comorbidity severity adjustment model to improve the prediction of in-hospital mortality among COPD patients using large-scale registry data from the Korea Disease Control and Prevention Agency.

**Method:**

This retrospective study used the Korea National Hospital Discharge In-Depth Injury Survey (KNHDIS) from 2011 to 2023, including 13,385 hospitalized COPD patients (ICD-10 code J44). All analyses accounted for the complex sample design with stratification, clustering, and sampling weights. The baseline model used multivariable logistic regression with conventional covariates, whereas the extended model incorporated comorbidity clusters derived from Apriori association rule mining to capture interconnected disease patterns. Odds ratios (OR) and 95% confidence intervals (CI) were calculated to assess determinants of in-hospital mortality.

**Result:**

In the baseline model, advanced age, emergency admission, larger hospital size, and higher CCI scores were significantly associated with increased mortality. In the extended model with Apriori-derived clusters, additional high-risk profiles were identified: (i) septicemia with pneumonia, (ii) respiratory failure with sequelae of tuberculosis, and (iii) pulmonary heart disease with sequelae of tuberculosis. The predictive performance improved with the inclusion of comorbidity clusters (AUC 0.753 vs. 0.732), indicating that the cluster-augmented model provided superior discrimination compared with the baseline model.

**Conclusion:**

These findings indicate that integrating data-driven comorbidity patterns into traditional risk models enhances mortality risk stratification for COPD inpatients and may support both clinical decision-making and the development of evidence-based health policies.

## Introduction

Chronic obstructive pulmonary disease is a leading cause of morbidity and mortality worldwide, imposing a substantial socioeconomic burden [1,2]. In 2015, the global prevalence of chronic obstructive pulmonary disease (COPD) among individuals aged 30 years or older was estimated between 250 million and 380 million, and COPD accounted for 2.6% of disability-adjusted life years (DALYs) worldwide in the same year [3–5]. COPD is defined by airflow limitation resulting from chronic bronchitis and emphysema, and manifests with respiratory symptoms including chronic cough, exertional dyspnea, sputum production, and wheezing [6,7]. Although COPD is not considered curable, early diagnosis and management can help mitigate symptoms and lower mortality risk [7,8]. Consequently, it is crucial to identify determinants of mortality associated with acute exacerbations in COPD patients, and implementing severity adjustment is essential for precise assessment and prediction of health outcomes.

Recently, a number of studies have aimed to identify predictive factors for mortality in COPD and to classify patient groups into clinical clusters [9–11]. Celli et al. identified four clinical parameters that predict the risk of death in COPD: body mass index (BMI), degree of airflow obstruction, dyspnea severity, and exercise capacity, leading to the development of the BODE index [9]. Another approach to categorizing COPD patients involves the use of cluster analysis, enabling the formation of homogeneous subgroups based on multiple disease-related variables [12]. Previous investigations have demonstrated that comorbid conditions play a significant role in hospitalization and mortality among COPD patients [13–15]. Multiple reports have found that COPD patients often experience a range of comorbidities, including cardiovascular disease, cerebrovascular disease, lung cancer, and diabetes. The Charlson Comorbidity Index (CCI) is extensively utilized for assessing comorbidities as predictors of mortality in these patients [16–21]. The CCI remains the standard tool for comorbidity adjustment, assigning a weight of 1–6 points for each of 19 predefined conditions identified through a review of medical records, and summing these values to produce a composite score [22]. The original CCI was validated on data from 685 breast cancer patients treated at a Connecticut hospital between 1962 and 1969. Subsequently, its application has been extended to various diseases and surgical procedures, where repeated studies have confirmed its validity [23]. Nevertheless, there is a recognized need for developing comorbidity indices tailored to specific diseases, regional characteristics, and contemporary advances in medical care, prompting ongoing research in this field [14,24,25].

A model that predicts health outcomes with greater accuracy can facilitate earlier targeted interventions for COPD patients and inform health policies that enhance patient outcomes. Comorbidities frequently occur in COPD patients and have been reported to influence both mortality and disease severity [26]. To reliably identify comorbidities, it is more effective to utilize data from community or national registries than cohort studies dependent on self-reported information. With the rising focus on big data in healthcare, there has been an increase in research using machine learning, deep learning, and artificial intelligence-based data mining methods [27–29]. Regarding analytical approaches, recent studies apply pattern analysis among comorbidities to uncover latent relationships, moving beyond traditional descriptive statistics [25]. The association rule technique, commonly used for comorbidity pattern analysis, is part of data mining and does not require traditional parametric statistical assumptions, such as normality or linearity [30]. Because it lacks a target variable, it is considered an unsupervised learning approach and is also referred to as market basket analysis in marketing as a strategy for discovering concealed associations among numerous variables [31]. Nevertheless, research specifically addressing comorbidity adjustment models for COPD patients using large-scale, validated registry data remains limited. Existing studies predominantly assess the predictive performance of established comorbidity adjustment models, such as CCI and ECI, within specific hospital populations or targeted groups [11,15,32,33].

Therefore, the aim of this study is to construct a comorbidity severity adjustment model to improve the prediction of mortality—a key health outcome—in COPD patients who bear a significant socioeconomic disease burden, utilizing large-scale registry data from the Korea Disease Control and Prevention Agency. The specific objectives are as follows. First, to characterize hospitalized COPD patients and identify determinants of mortality. Second, to evaluate comorbidity patterns among deceased COPD inpatients through association rule analysis. Third, to assess the predictive utility of the comorbidity severity adjustment model for COPD mortality.

## Methods

### Data source

This study involved a retrospective analysis using the Korea National Hospital Discharge Injury Survey (KNHDIS), a nationwide dataset compiled by the Korea Disease Control and Prevention Agency (KDCA) from 2011 to 2023. The KNHDIS, conducted annually since 2005, is recognized as a national official statistic by Statistics Korea (Approval No. 117060). The survey collects discharge medical records from general hospitals with a minimum of 100 beds, excluding single-specialty hospitals, throughout South Korea. To ensure representativeness and incorporate changes such as hospital openings or closures, facility type modifications, and adjustments in bed capacity, the sampling structure has been periodically updated [34]. The number of hospitals included in the survey expanded from 170 in 2008–2017, to 200 during 2018–2019, then to 220 in 2020–2021, and reached 250 from 2022 onward [34]. Such adjustments enable the dataset to accurately reflect regional variability and achieve a practical balance between statistical accuracy and operational feasibility. Employing a stratified two-stage cluster sampling design, hospitals served as primary sampling units and discharged patients as secondary units. Initially, 150 hospitals were sampled in 2005, with expansion to 250 hospitals by 2023, based on Neyman allocation. About 9% of all discharges in the selected hospitals were included. Each record features one principal diagnosis plus up to 20 secondary diagnoses, utilizing codes from the 10th edition of the International Classification of Diseases (ICD-10). The data comprise 30 variables, covering demographic and geographic attributes, admission and discharge information, clinical and treatment data, and details on external causes of injury. The KNHDIS database is widely used to generate national health statistics and inform injury-prevention strategies, and its methodological facilitates valid estimation of both national and regional indicators.

This study used de-identified, publicly available secondary data from the Korea National Hospital Discharge In-depth Injury Survey (KNHDIS), provided by the Korea Disease Control and Prevention Agency (KDCA). Access to the dataset requires submission of a formal request and approval from the KDCA. All data used in this study were accessed on December 24, 2024. Ethical approval was obtained from the Institutional Review Board of Dong-A University (IRB No. 2–1040709-AB-N-01–202506-HR-042–02), and the study was conducted in accordance with all relevant guidelines and regulations.

### Study population

**Fig 1:**
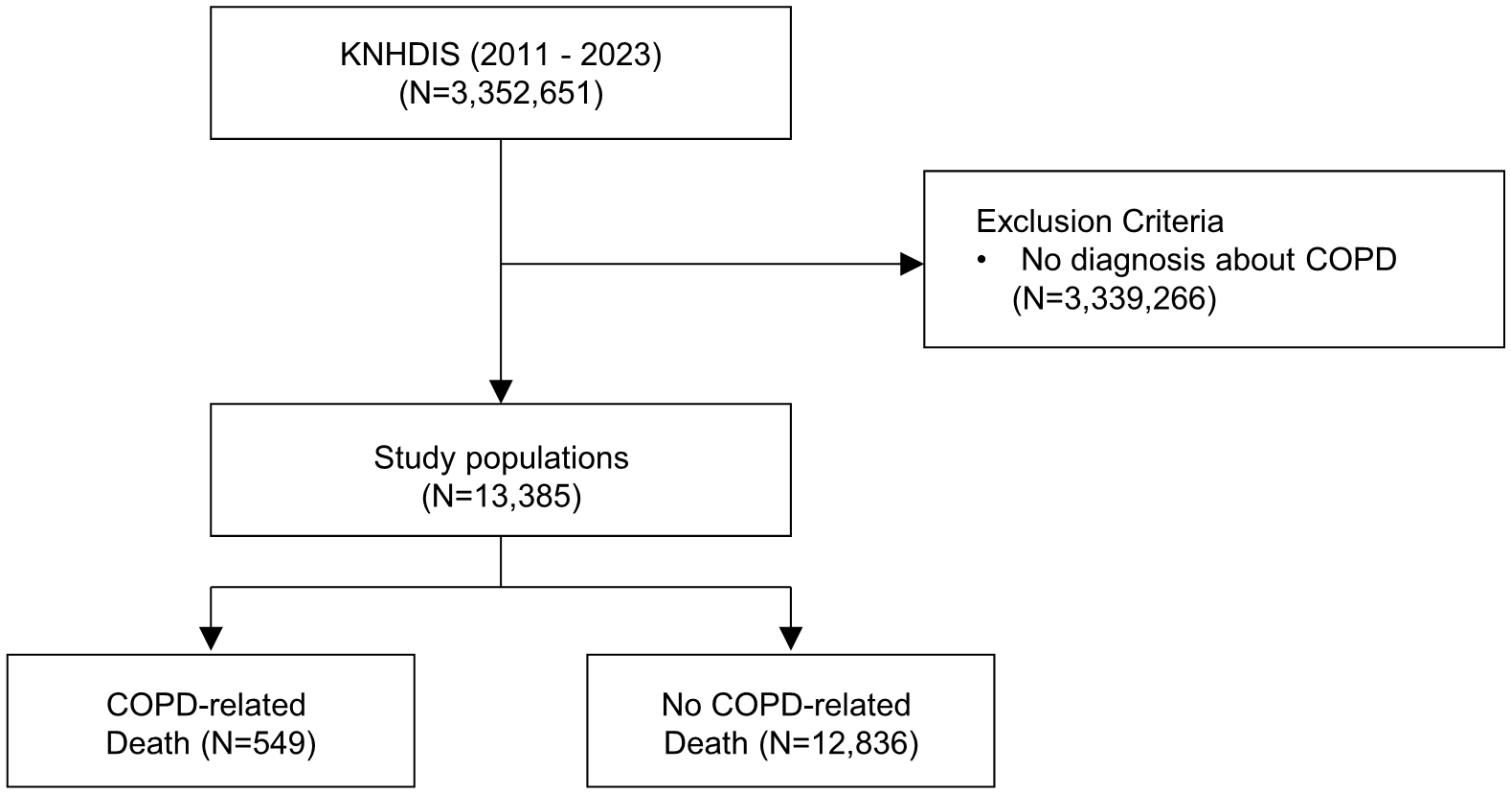
Study patient flow chart.

This retrospective study analyzed patients with chronic obstructive pulmonary disease (COPD), identified by ICD-10 code J44 and recorded as the principal discharge diagnosis. From the Korea National Hospital Discharge Injury Survey (KNHDIS), which included 3,352,651 discharge records, several exclusion criteria were applied. Records without a COPD diagnosis based on the principal were excluded to ensure diagnostic accuracy, and records with incomplete or missing data were excluded to ensure data validity. Following these procedures, a total of 13,385 patients were included in the final study cohort. These patients were subsequently divided into two groups according to in-hospital all-cause mortality (Fig 1). Informed consent was waived due to the retrospective design and the prior de-identification of all records.

### Study variables and outcomes

Collected variables included socio-demographic characteristics (sex, age) and hospital-level factors (admission route, length of stay [days], insurance type, bed size, hospital residence). Additionally, comorbidity burden was measured using the Charlson Comorbidity Index (CCI). Age was divided into four categories: younger than 65 years, 65–74 years, 75–84 years, and 85 years or older. For hospital region, three categories were used based on geographic accessibility and administrative similarity: Seoul/Gyeonggi, metropolitan cities, or provincial regions. Admission route, health coverage, and bed size were grouped for this analysis consistent with the KNHDIS variable structure. Comorbidities were assessed from up to 20 secondary ICD-10 diagnoses. Implausible values were treated as missing, and analyses were conducted using all available data. The primary outcome measured was all-cause in-hospital mortality.

### Charlson Comorbidity Index (CCI)

The Charlson Comorbidity Index (CCI) serves as a validated tool for evaluating comorbidity burden and for risk adjustment in outcome research [35,36]. Among the available ICD-10 coding algorithms, we selected the version developed by Quan et al. [24] and retained its original weighting system, as multiple validation studies have consistently demonstrated superior discrimination and model fit for both in-hospital and long-term mortality across diverse populations, outperforming alternative algorithms such as the approach proposed by Sundararajan et al. [11]. Accordingly, this study employed the Quan algorithm to improve the validity of comorbidity adjustment, as reflected by improved diagnostic accuracy and predictive performance when applied to administrative hospital data [24,37,38]. Each comorbidity was coded as present or absent, and the aggregate score was calculated using the designated weights. CCI scores were categorized into four strata (0, 1, 2, and ≥3), reflecting increasing severity of comorbidity.

### Statistical analysis

All statistical analyses accounted for the complex sampling design of the Korea National Hospital Discharge In-depth Injury Survey (KNHDIS). The KNHDIS employs a two-stage stratified cluster sampling design; therefore, analyses incorporated survey-design elements such as strata, clusters, and sampling weights. This approach ensured the attainment of nationally representative population estimates and valid variance calculations, in accordance with the 2023 KNHDIS utilization guidelines [35]. Categorical variables were summarized as frequencies (n) and weighted percentages (%), while group differences were examined using survey-adjusted chi-square tests. Continuous variables were analyzed through design-adjusted weighted mean comparisons, accounting for stratification and clustering. Before developing the mortality prediction models, multicollinearity among covariates was evaluated using VIF values. To investigate factors associated with in-hospital mortality, survey-weighted logistic regression models were fitted to estimate odds ratios (ORs) and 95% confidence intervals (CIs). Model 1 included socio-demographic and clinical covariates, while Model 2 additionally incorporated comorbidity clusters derived from Apriori-based association rule analysis to assess the impact of interconnected disease patterns on mortality risk. These analytical procedures were chosen to ensure methodological appropriateness for complex survey data and to enhance the accuracy and interpretability of population-level estimates. All analyses were conducted using SAS version 9.4 (SAS Institute, Cary, NC) and R version 4.4.1 (R Foundation for Statistical Computing, Vienna, Austria).

### Association rule analysis of comorbidities

We used association rule analysis to identify clinically significant patterns of comorbidities that co-occur among hospitalized COPD patients. This analytical technique is commonly employed to extract concealed associations from large-scale datasets, thereby facilitating clinical decision-making and refined stratification strategies [39,40]. Of the various association rule mining approaches, the Apriori algorithm is recognized as a foundational method [30] and is widely adopted in fields such as medicine and economics [37]. The algorithm identifies frequent item sets and assesses their relevance within the dataset using measures such as support, confidence, and lift [37]. While initially designed for market-basket analysis, Apriori has also proven effective in healthcare research for detecting diagnostic co-occurrences, delineating patient subgroups, and guiding precision-medicine interventions [39,41]. In this study, we specified the minimum confidence level, minimum support level, and minimum lift as 0.10, 0.02, and 1, respectively [38,42]. Support quantifies the frequency with which a combination of conditions arises across the entire dataset, indicating the proportion of records identified with both items. Confidence measures the likelihood of encountering one condition in the presence of another, reflecting the strength of their if-then association. Lift, taking 1 as the reference, assesses whether the co-occurrence of conditions exceeds what would be expected by chance; values above 1 denote a meaningful association [41]. Additionally, only association rules with a lift exceeding 1.0 were selected, ensuring the identification of clinically relevant patterns. The methodology enabled us to reveal predominant clusters of comorbid diseases, offering practical insights for risk assessment and the customization of management strategies. The Apriori analyses were performed using the arules package in R version 4.4.1.

## Result

### Descriptive statistics

Table 1 presents the baseline socio-demographic characteristics, hospital-level factors, and Charlson Comorbidity Index (CCI) scores among hospitalized COPD patients, stratified by all-cause in-hospital mortality. In the in-hospital mortality group, factors such as older age, emergency admission, extended length of hospital stay, larger hospital capacity, and greater comorbidity burden were consistently linked with higher in-hospital mortality (p < 0.001), while both insurance type and hospital geographic region showed statistically significant but less pronounced associations (p < 0.05). The data indicate an age-related increase in mortality risk. Specifically, a mortality rate of 7.1% was observed in patients aged 85 years or older, followed by 4.2% in those aged 75–84 years, 3.3% in those aged 65–74 years, and 1.9% in patients younger than 65 years, highlighting greater vulnerability with increasing age. The pattern of admission also showed notable differences: 6.3% of patients admitted through the emergency department died, compared to 1.9% of those admitted through the outpatient department. Analysis of insurance type identified differences related to socioeconomic status. Within the mortality group, 4.1% of patients were recipients of national health insurance, 2.8% were covered by medical aid, and 3.4% were insured under other categories. Hospital size was associated with differences in in-hospital mortality rates. Institutions with more than 1,000 beds reported the highest in-hospital mortality rate at 5.0 percent. This was followed by hospitals with 500–999 beds at 4.9 percent and those with 300–499 beds at 4.3 percent. The lowest mortality rate was observed in hospitals with 100–299 beds, at 3.1 percent. Analysis by region also revealed geographical disparities. The highest mortality was observed in hospitals located in Seoul and Gyeonggido, where the rate was 4.6 percent. Hospitals in provincial areas reported a rate of 3.6 percent, while those in metropolitan cities had the lowest rate at 3.4 percent. These findings suggest that both institutional and regional factors may influence patient outcomes. The mortality group also carried a greater comorbidity burden. Among these patients, 7.7 percent had a CCI score of three or above, compared with 3.3 percent who had a score of zero.

**Table 1:** Baseline Characteristics and Charlson Comorbidity Index(CCI) distribution among Hospitalized COPD Patients.

Variables	Category	In hospital Mortality				*χ* ^2^ *or t*	*p*
		N		Weighted %			
		No	Yes	No	Yes		
Sex	Male	9,774	427	96.2	3.8	0.057	0.811
	Female	3,062	122	96.3	3.7		
Age	< 65	2,772	60	98.1	1.9	22.416	<0.001
	65 ~ 74	3,860	146	96.7	3.3		
	75 ~ 84	4,873	242	95.8	4.2		
	≥ 85	1,331	101	92.9	7.1		
Admission route	Emergency	6,095	412	93.7	6.3	91.269	<0.001
	Outpatient	6,735	137	98.1	1.9		
	Other	6	0	100	0		
Length of Stay(day)		11.60 ± 0.18	22.92 ± 1.73			−6.515	<0.001
Insurance type	Health Insurance	10,204	458	95.9	4.1	4.089	0.017
	Medical	2,433	84	97.2	2.8		
	Other	199	7	96.6	3.4		
Bed size	100-299	4,492	147	96.9	3.1	7.892	<0.001
	300-499	1,715	74	95.7	4.3		
	500-999	5,209	256	95.1	4.9		
	≥ 1000	1,420	72	95.0	5.0		
Hospital residence	Seoul Gyeonggido	3,460	158	95.4	4.6	3.340	0.036
	Metropolitan city	3,453	151	96.6	3.4		
	Province	5,923	240	96.4	3.6		
CCI Index	0	9,192	324	96.7	3.3	13.838	<0.001
	1	2,357	120	95.7	4.3		
	2	822	64	93.3	6.7		
	≥ 3	465	41	92.3	7.7		

### Comorbidities

Table 2 presents the distribution of comorbidities, as defined by the Charlson Comorbidity Index (CCI), among patients hospitalized with COPD, stratified according to all-cause in-hospital mortality. Categorical variables were analyzed using complex-sample survey-weighted chi-square tests. The highest in-hospital mortality rate was observed in patients with metastatic solid tumor at 14.2% (p = 0.001), followed by those with myocardial infarction at 13.1% (p < 0.001). Cerebrovascular disease and any malignancy, including lymphoma and leukemia, each demonstrated mortality rates of 7.3%, with the association for cerebrovascular disease reaching statistical significance (p = 0.004). Patients with congestive heart failure had a mortality rate of 7.1% (p < 0.001), those with renal disease 6.9% with a significant association (p = 0.003), and patients with diabetes without chronic complications 4.2% (p < 0.001). These findings suggest that specific comorbidities, such as metastatic solid tumor, cerebrovascular disease, and renal disease, are significantly associated with increased in-hospital mortality among hospitalized COPD patients.

**Table 2:** Distribution of comorbidities by Charlson Comorbidity Index (CCI) in COPD Patients.

Variables	In-hospital Mortality				*χ* ^2^	*p*
	N		Weighted %			
	No	Yes	No	Yes		
Acute Myocardial Infarction	95	14	86.9	13.1	18.701	<0.001
Chronic Congestive Heart Failure	755	63	92.9	7.1	21.572	<0.001
Peripheral Arterial Disease	136	11	94.2	5.8	1.580	0.209
Cerebrovascular Disorders	278	22	92.7	7.3	8.189	0.004
Neurocognitive Disorders	254	13	95.9	4.1	0.053	0.818
Inflammatory Rheumatologic Diseases	51	3	91.0	9.0	2.061	0.151
Peptic Ulcer Disorders	135	12	94.0	6.0	2.160	0.142
Mild Hepatic Disease	318	14	96.7	3.3	0.238	0.625
Diabetes Mellitus without Chronic Complications	1,528	72	95.8	4.2	18.701	<0.001
Diabetes Mellitus with Chronic Complications	160	12	94.6	5.4	1.105	0.293
Hemiplegia or Paraplegia	25	3	89.3	10.7	3.267	0.071
Chronic Renal Disease	322	30	93.1	6.9	9.025	0.003
Any Malignancy, Including Lymphoma or Leukemia	476	36	92.7	7.3	13.006	<0.001
Moderate or Severe Advanced Liver Disease	9	1	90.1	9.9	1.010	0.315
Metastatic Solid Tumor	61	7	85.8	14.2	11.525	0.001
AIDS/HIV	2	0	100	0	0.078	0.780

### Association rule mining

**Fig 2:**
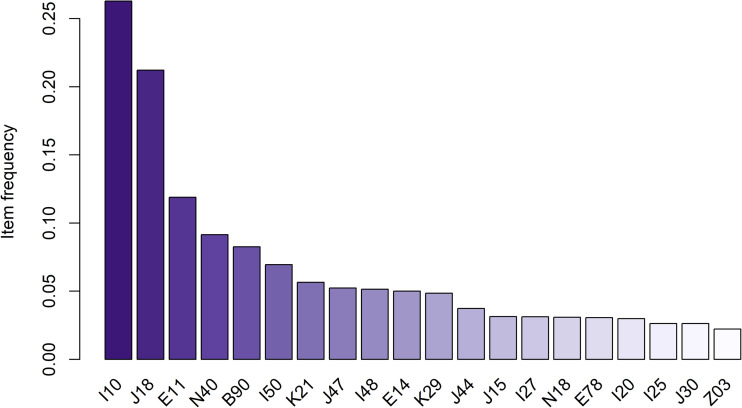
Frequent comorbidities observed among hospitalized COPD patients. Note. The Y-axis represents item frequency (relative), and the X-axis lists ICD-10 diagnostic categories; ICD-10 diagnostic categories:10 Essential (primary) hypertension; J18 Pneumonia, organism unspecified; E11 Type 2 diabetes mellitus; N40 Hyperplasia of prostate; B90 Sequelae of tuberculosis; I50 Heart failure; K21 Gastro-oesophageal reflux disease; J47 Bronchiectasis; I48 Atrial fibrillation and flutter; E14 Unspecified diabetes mellitus; K29 Gastritis and duodenitis; J44 Other chronic obstructive pulmonary diseases; J15 Bacterial pneumonia, NEC; I27 Other pulmonary heart diseases; N18 Chronic kidney disease; E78 Disorders of lipoprotein metabolism and other lipidemias; I20 Angina pectoris; I25 Chronic ischemic heart disease; J30 Vasomotor and allergic rhinitis; Z03 Medical observation and evaluation for suspected diseases and conditions, ruled out.

**Fig 3:**
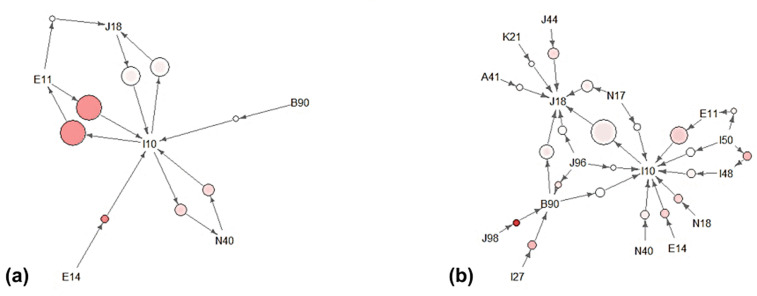
Network visualization of comorbidity association rules generated by the Apriori algorithm. (a) Overall study population; (b) In-hospital mortality cohort Note. ICD-10 diagnostic categories: E11 Type 2 diabetes mellitus; J18 Pneumonia, organism unspecified; I10 Essential (primary) hypertension; B90 Sequelae of tuberculosis; N40 Hyperplasia of prostate; E14 Unspecified diabetes mellitus; A41 Other sepsis; K21 Gastro-oesophageal reflux disease; J44 Other chronic obstructive pulmonary diseases; J96 Respiratory failure, not elsewhere classified; J98 Other respiratory disorders; I27 Other pulmonary heart diseases; N18 Chronic kidney disease; I48 Atrial fibrillation and flutter; I50 Heart failure; N17 Acute renal failure.

Table 3 presents the association rules derived from Apriori-based analysis of comorbid diagnosis patterns among hospitalized COPD patients. The association rules fulfilled the pre-established thresholds for support, confidence, and lift as described in the Methods section, ensuring that only clinically relevant patterns were included [42,62]. Analyses were conducted separately for the entire study cohort and the subgroup of patients who died during their hospital stay. A visual overview of the top 20 comorbidity association rules derived from Apriori algorithm is shown in Fig 2. In the overall population, three association rules were recognized as the most notable. The co-occurrence of unspecified diabetes mellitus (E14) and essential hypertension (I10) (support = 0.026, confidence = 0.511, lift = 1.942) indicated a nearly twofold increased likelihood compared to chance. The combination of type 2 diabetes mellitus (E11) and essential hypertension (I10) (support = 0.059, confidence = 0.496, lift = 1.888) demonstrated a similar elevation in joint occurrence. Additionally, prostatic hyperplasia (N40) with essential hypertension (I10) (support = 0.033, confidence = 0.357, lift = 1.359) emerged as a relevant association pattern. Within the in-hospital mortality subgroup, 21 distinct rules were identified (Fig 3). Among the most significant was the co-occurrence of other respiratory disorders (J98) and sequelae of tuberculosis (B90) (support = 0.023, confidence = 0.647, lift = 4.971), representing the strongest association observed. Congestive heart failure (I50) and atrial fibrillation (I48) (support = 0.029, confidence = 0.264, lift = 3.044) were also prominent. Pulmonary heart disease (I27) together with sequelae of tuberculosis (B90) (support = 0.031, confidence = 0.395, lift = 3.033) formed another key pattern. The pairing of unspecified diabetes mellitus (E14) and essential hypertension (I10) (support = 0.031, confidence = 0.600, lift = 2.400) remained notable. Respiratory failure (J96) with sequelae of tuberculosis (B90) (support = 0.030, confidence = 0.371, lift = 2.224) was also frequently observed. Several additional rules were also identified, including chronic kidney disease (N18) with essential hypertension (I10) (support = 0.031, confidence = 0.556, lift = 2.222), prostatic hyperplasia (N40) with essential hypertension (I10) (support = 0.029, confidence = 0.400, lift = 1.600), tuberculosis (A15) with pneumonia (J18) (support = 0.023, confidence = 0.478, lift = 1.386), and gastro-esophageal reflux disease (K21) with pneumonia (J18) (support = 0.021, confidence = 0.500, lift = 1.449). These rules represent additional multimorbidity clusters of potential clinical importance and novelty beyond those most strongly associated with in-hospital mortality.

**Table 3:** Apriori-Derived Comorbidities Association Rules in COPD Patients.

No	Rules	n	Support	Confidence	Lift
**The overall study cohort**					
1	E14 → I10	264	0.026	0.511	1.942
2	E11 → I10	610	0.059	0.496	1.888
3	N40 → I10	338	0.033	0.357	1.359
**In-hospital Mortality = Yes (in-hospital death)**					
1	J98 → B90	11	0.023	0.647	4.971
2	I50 → I48	14	0.029	0.264	3.044
3	I27 → B90	15	0.031	0.395	3.033
4	E14 → I10	15	0.031	0.600	2.400
5	J96 → B90	11	0.023	0.289	2.224
6	N18 → I10	15	0.031	0.556	2.222
7	E11 → I10	30	0.062	0.517	2.069
8	J44 → J18	19	0.039	0.655	1.899
9	N40 → I10	14	0.029	0.400	1.600
10	I50 → E11	10	0.021	0.189	1.574
11	K21 → J18	10	0.021	0.500	1.449
12	I48 → I10	15	0.031	0.357	1.429
13	A41 → J18	11	0.023	0.478	1.386
14	N17 → J18	20	0.041	0.455	1.317
15	B90 → J18	25	0.052	0.397	1.150
16	J96 → J18	15	0.031	0.395	1.144
17	N17 → I10	12	0.025	0.273	1.091
18	I50 → I10	14	0.029	0.264	1.057
19	I10 → J18	44	0.091	0.364	1.054
20	J96 → I10	10	0.021	0.263	1.053
21	B90 → I10	16	0.033	0.254	1.016

**Note**: Association rules were identified via the Apriori algorithm. Support, confidence, and lift metrics quantified rule strength; a minimum support threshold of 0.02 and a lift of 1.0 were applied. hospitalization.

### Adjusted outcomes

Table 4 presents the results of multivariable logistic regression for the outcome of in-hospital mortality in COPD patients, utilizing a complex sample design. Adjusted odds ratios (OR) with 95% confidence intervals (CI) are shown. A baseline model with conventional predictors (Model 1) was compared with an expanded model incorporating Apriori-derived comorbidity clusters (Model 2).

**Table 4:** Complex sample logistic regression results for in-hospital mortality (Model 1).

Variables		Estimate	SE	p	OR (95% CI) (Lower-Upper)
Sex	Female				
	Male	0.06	0.13	0.609	1.07(0.83-1.36)
Age	<65				
	65-74	0.48	0.18	<0.01	1.62(1.14-2.30)
	75-84	0.73	0.17	<0.001	2.07(1.49-2.90)
	≥85	1.28	0.19	<0.001	3.58(2.46-5.20)
Admission route	emergency				
	outpatient	−1.13	0.12	<0.001	0.32(0.26-0.41)
	other	−10.62	0.46	<0.001	0.00(0.00-0.00)
Length of stay (day)		0.01	0.00	<0.01	1.01(1.01-1.02)
Insurance type	others				
	health insurance	−0.06	0.43	0.888	0.94(0.41-2.19)
	medical	−0.33	0.45	0.454	0.72(0.30-1.72)
Bed size	100-299				
	300-499	0.26	0.16	0.109	1.29(0.94-1.77)
	500-999	0.25	0.12	<0.05	1.28(1.01-1.62)
	≥1000	0.19	0.17	0.253	1.21(0.87-1.69)
Hospital residence	province				
	Seoul Gyeonggi-do	0.08	0.13	0.569	1.08(0.83-1.40)
	metropolitan city	−0.16	0.13	0.204	0.85(0.67-1.09)
CCI_Index	0				
	1	0.23	0.13	0.072	1.26(0.98-1.61)
	2	0.60	0.17	<0.001	1.83(1.32-2.54)
	≥3	0.62	0.22	<0.01	1.85(1.21-2.83)

**Note**. Findings are obtained from complex sample logistic regression (Model 1).

In the Complex Sample Logistic Regression Model of In Hospital Mortality (Model 1), older age emerged as a robust independent predictor of mortality. Relative to patients under 65 years, those aged 65–74 years had an OR of 1.62 (95% CI: 1.14–2.30), those aged 75–84 years had an OR of 2.07 (95% C: I 1.49–2.90), and individuals aged 85 years or older exhibited the highest risk with an OR of 3.58 (95% CI: 2.46–5.20). Admission via the outpatient department correlated with a reduced risk compared to emergency admission, with an OR of 0.32 (95% CI: 0.26–0.41). Patients hospitalized in institutions with 500–999 beds had a slightly elevated risk compared to those in hospitals with 100–299 beds, with an OR of 1.28 (95% CI: 1.01–1.62). An increased Charlson Comorbidity Index (CCI) was also linked to higher mortality risk, CCI of 2, 1.83 (95% CI: 1.32–2.54), and CCI of 3 or higher, 1.85 (95% CI: 1.21–2.83), both compared to a CCI of 0.

**Fig 4:**
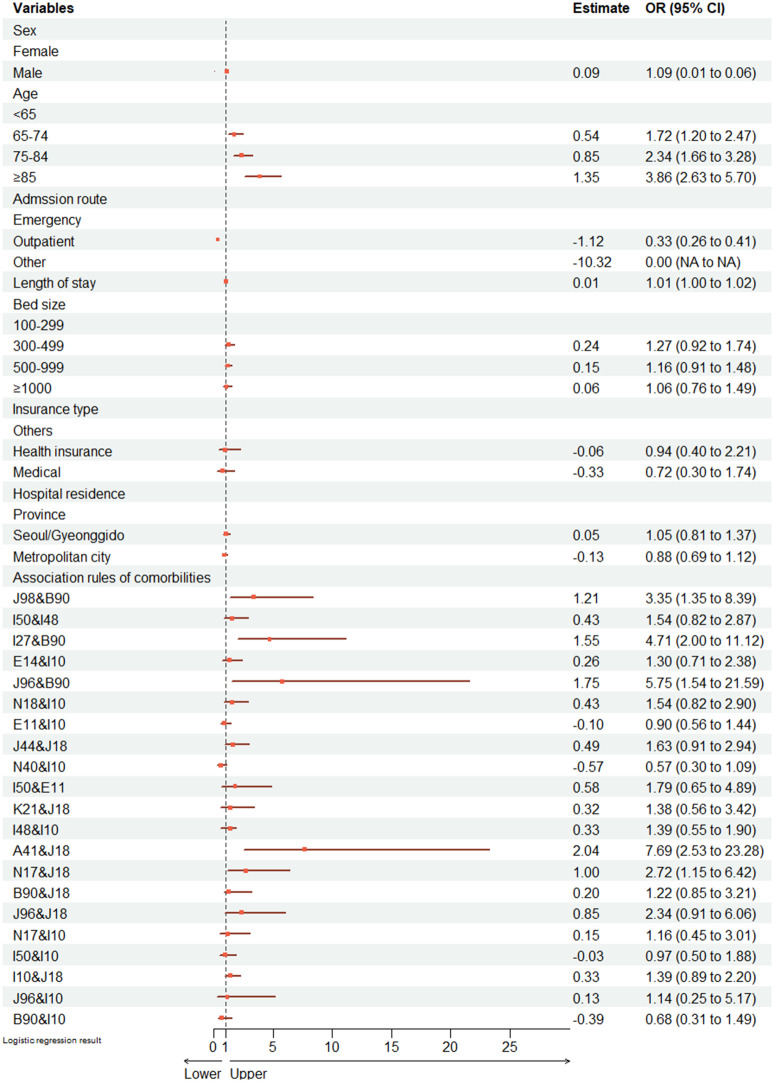
Forest plot for Model 2: Mortality-associated comorbidities identified through Apriori analysis. Note: Odds ratios (ORs) and 95% confidence intervals (CIs) were estimated using complex sample logistic regression. Model 2 incorporates both general characteristics and Apriori-derived comorbidities. The ICD-10 diagnostic descriptions for the Apriori-derived comorbidities included in this forest plot are provided in the legends of Figs 2 and 3.

**Fig 5:**
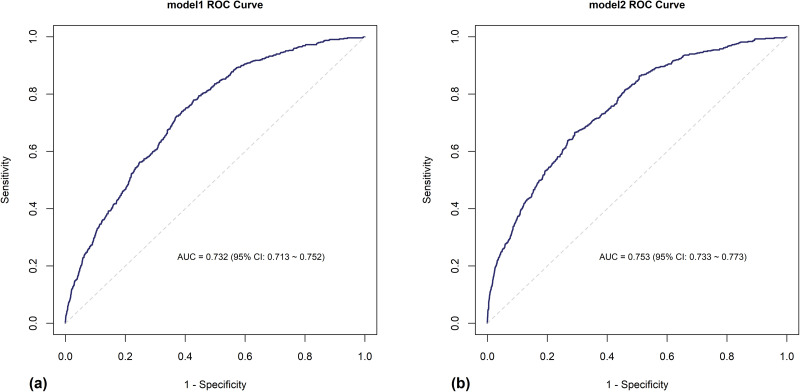
ROC curve comparison of logistic regression models for in-hospital mortality: (a) CCI-based (Model 1), (b) Apriori-derived comorbidity-based (Model 2). **Note.** Model 1 comprises general characteristics and Charlson Comorbidity Index (CCI) variables, and Model 2 comprises general characteristics and comorbidities detected using Apriori association rule analysis. Complex sample logistic regression was performed for both models. AUC = area under the ROC curve.

After integrating Apriori‑derived comorbidity clusters (Model 2), the relationships for conventional predictors such as age and admission route were consistent with those observed in Model 1. Moreover, several Apriori‑derived clusters were significantly and independently related to in‑hospital mortality. For example, septicemia with pneumonia (A41 & J18) revealed an OR of 7.61 (95% CI: 2.53–23.05), respiratory failure with sequelae of tuberculosis (J96 & B90) had an OR of 5.75 (95% CI: 1.53–21.44), and pulmonary heart disease with sequelae of tuberculosis (I27 & B90) had an OR of 4.76 (95% CI: 2.02–11.29) (Fig 4). Compared to the baseline, model metrics improved after adding Apriori‑derived clusters. Model 1 reported an AUC of 0.732 (95% CI: 0.712–0.752), AIC 4030.71, and Hosmer and Lemeshow 0.082, while Model 2 yielded an AUC of 0.753 (95% CI: 0.733–0.773), AIC 3997.68, and Hosmer and Lemeshow 0.098 (Fig 5). These results demonstrate that integrating Apriori-derived comorbidity patterns with conventional predictors modestly improved the model's discriminative ability and effectiveness in risk stratification for in-hospital mortality among COPD patients.

## Discussion

In this study, we developed a comorbidity severity adjustment model to improve the prediction of in-hospital mortality among patients with chronic obstructive pulmonary disease (COPD) using large-scale registry data from the Korea Disease Control and Prevention Agency (KDCA). Our specific objectives were threefold: first, to characterize hospitalized COPD patients and identify factors associated with in-hospital mortality; second, to analyze comorbidity patterns among deceased COPD inpatients using association rule analysis; and third, to evaluate the predictive performance of a comorbidity severity adjustment model that integrates conventional risk factors with data-driven comorbidity patterns.

In addition to traditional socio-demographic and health utilization variables, we applied the Apriori algorithm, an unsupervised learning technique, to uncover novel comorbidity patterns not previously reported in COPD research. Older age emerged as the most significant determinant of mortality risk in multivariable models. A previous cross-sectional analysis reported an adjusted odds ratio of 2.27 for mortality among patients aged 79 years and older [17], and several cohort studies have consistently identified advanced age as a strong predictor of in-hospital mortality in COPD patients [41,43,44]. COPD patients admitted through the emergency department exhibited the highest in-hospital mortality rate at 6.3% compared to all other admission pathways. This rate exceeds the 4.9% documented in the DECAF score validation study [45], likely due to our broader inclusion criteria, which considered all emergency admissions irrespective of exacerbation severity or specific clinical parameters. Supporting this finding, a French cohort study [34] revealed that dyspnoea severity, older age, and clinical severity indicators independently predicted in-hospital mortality for COPD patients admitted via emergency services. These results suggest that emergency admission serves not just as a hospital entry point but may also reflect acute physiological deterioration, highlighting the necessity for early risk assessment and targeted interventions upon admission.

The mean length of stay (LOS) for COPD patients who died during hospitalization was 22.9 days. Each additional day of hospitalization correlated with increased odds of in-hospital mortality (OR: 1.010). In contrast, a previous cohort study [46] reported a shorter mean LOS of 8.6 days for patients who died within one year post-discharge and found that a longer LOS was associated with heightened odds of post-discharge mortality (OR: 1.024; 95% CI: 0.996–1.053). Despite variations in study design and population, both studies consistently indicate that prolonged LOS correlates with an elevated mortality risk in COPD. Collectively, these observations suggest that LOS may be a clinically relevant prognostic indicator for COPD mortality.

A clear association was observed between the Charlson Comorbidity Index (CCI) and in-hospital mortality, with higher CCI scores consistently associated with increased mortality risk [17,32,47]. Compared with patients with a CCI score of 0, adjusted odds ratios were 1.26 for CCI 1, 1.83 for CCI 2, and 1.85 for CCI 3 or higher. These findings are consistent with previous large-scale studies reporting a robust dose–response relationship between comorbidity burden and mortality [17], which aligns with the present study’s results. Furthermore, data from a prospective, multicenter observational study indicated that approximately 40% of COPD patients died during follow-up, with higher CCI scores strongly associated with poorer long-term survival [14].

We analyzed comorbidity patterns related to CCI across different mortality outcomes by comparing the disease profiles of deceased and surviving patients. Our dataset revealed significant differences in the prevalence rates of various conditions: metastatic solid tumors (14.2%), myocardial infarction (13.1%), cerebrovascular disease and any malignancy, including lymphoma and leukemia (7.3%), congestive heart failure (7.1%), renal disease (6.9%), and diabetes without chronic complications (4.2%) between those who died and those who survived. These findings align with previous studies [44], which reported notably higher rates of cerebrovascular disease (26.0%), hypertension (42.7%), atrial fibrillation (26.0%), and chronic kidney disease (15.6%) in the mortality group compared to the survivor group. Additionally, a national cross-sectional study [48] found that among individuals with COPD, malignant neoplasms of the bronchus and lung had the highest adjusted incidence rate ratio of 2.51, followed by heart failure (1.38), cerebrovascular disease (1.05), and ischemic heart disease (1.05), underscoring the significant impact of these comorbidities on mortality risk [48].

Our Apriori-based association rule mining further supported these findings by identifying frequent co-occurrences of comorbidities such as lung cancer, pneumonia, diabetes, heart failure, and tuberculosis sequelae among deceased patients with COPD. Notably, we identified associations between hyperplasia of the prostate (N40) and essential (primary) hypertension (I10), representing chronic disease patterns predominantly observed in male patients within the mortality group. In addition, gastro-oesophageal reflux disease (K21) frequently co-occurred with pneumonia (J18), suggesting that the combination of GERD and unspecified bacterial pneumonia may be associated with an increased risk of mortality. In the association rule analysis, unspecified diabetes mellitus (E14) paired with essential hypertension (I10) demonstrated a confidence level of 0.60, while type 2 diabetes mellitus (E11) combined with hypertension yielded a confidence of 0.517. Confidence reflects the conditional probability that one condition occurs given the presence of another, and a lift value greater than 1 indicates a meaningful positive association between comorbidities [49]. These findings indicate that specific comorbidity combinations among patients who died from COPD differ significantly from those observed in survivors. Some of the identified disease combinations, such as tuberculosis sequelae (B90) co-occurring with pulmonary heart disease (I27), have rarely been reported in previous COPD studies. While Divo et al. (2012) identified cardiovascular disease and cancer as leading contributors to mortality in COPD, our findings highlight a previously underrecognized interaction between tuberculosis sequelae and pulmonary heart disease [44]. These complex comorbidity patterns may confer a higher mortality risk than isolated conditions, underscoring their potential relevance for mortality risk stratification.

The mortality prediction model developed in this study incorporates age, CCI score, route of admission, and key comorbidity groupings identified through Apriori-based association rule mining. This model shows improved discriminatory capability (AUC = 0.753) compared to the conventional model that uses only the CCI score (AUC = 0.732). This suggests that an integrative approach utilizing multiple predictive variables may offer a more effective tool for clinical decision-making than models based on a single metric. Furthermore, the use of Apriori-based pattern discovery in this research represents a shift towards unsupervised learning methodologies capable of revealing underlying risk patterns, which differs from traditional regression approaches. Understanding the complex relationships among comorbidities enhances the foundational knowledge of Clinical Decision Support Systems (CDSS) [49] and supports the development of patient-centric interventions that can improve care quality through tailored treatment plans [50]. Additionally, these findings have direct relevance for preventive medicine, as they facilitate early identification of high-risk patients, inform targeted screening strategies, and support the integration of personalized risk information into Personal Health Record (PHR) systems.

This study has several limitations. First, its cross-sectional design prevents determining the temporal sequence between COPD and its major predictors, which limits causal interpretations. Second, the dataset did not include essential prognostic variables for elderly patients, such as socioeconomic status, daily functional independence, and polypharmacy. The absence of these factors may decrease the model’s accuracy in capturing the complex risk landscape for adverse outcomes in older adults. Third, the lack of clinical biomarkers, including laboratory findings or imaging results, made it difficult to evaluate disease severity with precision. However, this study benefits from the use of expert-validated ICD-10 codes sourced from a national health insurance claims database, enabling a comprehensive analysis of comorbidity and disease severity related to mortality risk in COPD patients. The extensive scale and consistent formatting of this large dataset enhance the relevance of our results for public health applications and demonstrate their potential for real-world translation. Future research should include subgroup analyses stratified by age and sex to better understand which specific comorbidity patterns are most clinically relevant, especially among elderly male patients. Additionally, incorporating a wider range of clinical indicators and increasing diversity within patient populations would further enhance the external validity and applicability of the study findings.

## Conclusions

This study identified distinct mortality-associated comorbidity patterns among hospitalized patients with COPD by utilizing expert-coded national claims data and applying Apriori-based association rule mining. By combining established predictors such as age and CCI score with data-derived comorbidity groupings, we created an improved predictive model that surpasses the performance of models based only on CCI. The study also evaluated whether this data-mining–based approach offers additional predictive value for COPD populations and assessed the generalizability and effectiveness of the model in nationally representative Korean data. Furthermore, integrating modifiable comorbidity patterns into predictive frameworks may enable the tailoring of interventions and inform public health prevention strategies at the national level, especially for high-risk individuals with chronic respiratory diseases.
